# Complete Genome Sequences and Methylome Analysis of Two Environmental *Spirochaetes*

**DOI:** 10.1128/MRA.00236-20

**Published:** 2020-04-09

**Authors:** Alexey Fomenkov, Margarita Y. Grabovich, Galina Dubinina, Natalia Leshcheva, Natalia Mikheeva, Tamas Vincze, Richard J. Roberts

**Affiliations:** a New England Biolabs, Inc., Ipswich, Massachusetts, USA; b Department of Biochemistry and Cell Physiology, Voronezh State University, Voronezh, Russia; c Federal Research Centre Fundamentals of Biotechnology of the Russian Academy of Sciences, Moscow, Russia; Portland State University

## Abstract

Here, we report the finished closed genomes of two environmental bacteria, Oceanispirochaeta crateria K2 and Thiospirochaeta perfilievii P (formally known as Spirochaeta perfilievii P). In addition, we provide methylation data and the associated enzymes predicted and confirmed to be responsible for each modified motif.

## ANNOUNCEMENT

Oceanispirochaeta crateria K2 and Thiospirochaeta perfilievii P were previously described (1) and deposited into the DSMZ collection under DSM 16308^T^ and DSM 19205^T^, respectively. They are particularly interesting because of their ability to metabolize sulfur, and ongoing biochemical studies are probing this feature of their metabolism. Methylome analysis was carried out as part of our ongoing studies of bacterial methylation. Genomic DNAs (10 μg) of both strains were obtained directly from the DSMZ collection.

Single-molecule real-time (SMRT) libraries were sequenced using the Pacific Biosciences (PacBio) RS II sequencing platform. Briefly, SMRTbell libraries were constructed from a genomic DNA sample sheared to ∼10 to 20 kb using the G-TUBE protocol (Covaris, Woburn, MA, USA), end repaired, and ligated to PacBio hairpin adapters. Incompletely formed SMRTbell templates and linear DNAs were digested with a combination of exonuclease III and exonuclease VII (New England Biolabs, Ipswich, MA, USA). DNA qualification and quantification were performed using the Qubit fluorometer (Invitrogen, Eugene, OR) and 2100 Bioanalyzer (Agilent Technologies, Santa Clara, CA). Two 8-kb SMRTbell libraries for each DNA species were prepared according to the PacBio sample preparation protocol, including additional separation on a BluePippin system (Sage Science, Beverly, MA), and were sequenced with C4-P6 chemistry on two SMRT cells, one with a non-size-selected (8-kb) library and one with a size-selected (10-kb) library, with a 360-minute collection time for each library. The total size-selected and non-size-selected sequencing reads (92,726 and 139,998 reads, respectively) with mean subread lengths of 10,188 and 9,090 bp, respectively, yielded 0.94 and 1.2 Gb of data for *T. perfilievii* P and *O. crateria* K2, respectively. They were *de novo* assembled using HGAP_Assembly.3 version 2.3.0 with default quality and read-length parameters and polished 3 times using Quiver (2). The polished assemblies generated 2 closed circular genome elements for *T. perfilievii* P with 32.62% GC content for the main chromosome (3,667,739 bp) and 29.80% GC content for the plasmid pSpeP (32,844 bp). The polished assembly generated one closed circular element for *O. crateria* K2 with 43.24% GC content for the main chromosome (4,012,482 bp). In all cases, direct repeats on the left and right flanks of the chromosomes and plasmid allowed closure as circular elements. The assembled sequences were annotated using the NCBI Prokaryotic Genome Annotation Pipeline (PGAP) (3, 4).

One advantage of the PacBio sequencing platform is its ability to detect the epigenetic state of sequenced DNA (4–7). Six m6A-modified DNA motifs were detected using SMRT motif and modification analysis version 2.3.0 in *T. perfilievii* P, and four DNA modification motifs were detected in *O. crateria* K2, where three of them were m6A and one motif contained m4C. Results are presented in Table 1, showing that most of the motifs were matched with the responsible methyltransferases (MTases), and have been deposited in REBASE (8).

**TABLE 1:** Summary of genome elements, methyltransferase genes, and their motifs identified in Thiopirochaeta perfilievii P and *Oceanispirochaeta crateria* K2

Species and genetic element	GenBank accession no.	Genome size (bp)	Genome coverage (×)	Methylase (RM^*e*^ system) name	Recognition motif^*a*^	Methylation RM type
*T. perfilievii* P						
Chromosome	CP035807	3,667,739	176.62	M.Spe19205I	RA**A**TTY	6mA, II
				M.Spe19205II	ATTA**A**T	m6A, II
				M.Spe19205II^*b*^^,^^*c*^	TAT**A**YN5GTG	m6A, I
				Spe19205IV^*c*^	GG**A**CY	m6A, II
				M1.Spe19205IV^*c*^	GG**A**CY	m6A, II
				M2.Spe19205IV^*c*^	GG**A**CY	m6A, II
				M.Spe19205V^*c*^	TTATA**A**	m6A, II
				Not assigned	VGA**A**GC	m6A, II
				M.Spe19205ORF800P^*c*^	Not active	III
Plasmid pSpeP	CP035808	32,844	259.1	M.Spe19205ORF17090P^*c*^	Not active	m6A, II
				M.Spe19205ORF17095P	Frameshift	m5C, II
*O. crateria* K2						
Chromosome	CP036150	4,012,482	125.33	M. OcrK2I	GGAT**C**C	m4C, II
				OcrKI2II	RA**A**TTY	m6A, II
				M.OcrK2III^*c*^	ATTA**A**T	m6A, II
				M.OcrK2IV^*c*^	TTATA**A**	m6A, II
				M.OcrK2V^*d*^	G**C**NGC	m5C, II

a Modified bases and the base opposite to them are in bold and underlined, respectively.

b This system requires the S1 subunit to determine specificity.

c MTase genes cloned and expressed in Escherichia coli strains ER2796 (9) and ER3081.

d The specificity of this MTase was established based on susceptibility of its genomic DNA to digestion with 5mC-specific enzymes (10).

e RM, restriction modification.

### Data availability.

The complete genome sequence of Thiospirochaeta perfilievii P is available in GenBank under the accession numbers CP035807 and CP035808. The original sequence reads have been deposited in NCBI under the SRA numbers SRR8551239 and SRR8551240. The BioProject number is PRJNA521375. The complete genome sequence of *Oceanispirochaeta crateria* K2 is available in GenBank under the accession number CP036150. The original sequence reads have been deposited in NCBI under the SRA numbers SRR8580034 and SRR8580035. The BioProject number is PRJNA521952.
